# Rabbit hunter uveitis: case report of tularemia uveitis

**DOI:** 10.1186/s12886-016-0332-z

**Published:** 2016-09-01

**Authors:** Céline Terrada, Said Azza, Bahram Bodaghi, Phuc Le Hoang, Michel Drancourt

**Affiliations:** 1 Ophtalmology Department, Assistance Publique-Hôpitaux de Paris, Paris, France; 2 Unité de Recherche sur les Maladies Infectieuses et Tropicales Emergentes (URMITE) UMR CNRS 6236 IRD 198, Méditerranée Infection, Aix-Marseille-Université, 27 Boulevard Jean Moulin, 13385 Marseille Cedex 5, France

**Keywords:** Tularemia, Uveitis, *Francisella tularensis*, Parinaud’s syndrome

## Abstract

**Background:**

Literature reports on ophthalmological manifestations related to tularemia, a zoonose caused by the bacterium *Francisella tularensis*, largely refer to Parinaud’s oculoglandular syndrome, which consists of the association of conjunctivitis with preauricular lymphadenitis. In this paper, we report a case of intraocular inflammation during tularemia infection.

**Case presentation:**

A 52-year-old Caucasian man was diagnosed with unilateral uveitis. The uveitis was posterior, with a 2+ vitritis and a large yellowish lesion involving the macula with an overlying sub-retinal detachment, extending inferiorly, and subretinal hemorrhages. Fluorescein angiography showed a late hyperfluorescence with focal vascular leakage. Ultrasound biomicroscopy confirmed the presence of a 3.8 mm parietal granuloma with a few calcifications in the left eye. While extensive work-up eliminated any other infectious and non-infectious etiology, tularemia was diagnosed by advanced serology consisting of two-dimensional Western-immunoblotting. The patient, a hunter, recalled having killed rabbits in the days before the symptoms appeared. Uveitis was rapidly controlled following treatment with doxycycline, yet three years after initiation of the treatment, the patient still complained of loss of vision in the left eye with a central scotoma.

**Conclusions:**

Posterior uveitis may be an infrequent manifestation of tularemia infection, and therefore this infection should be considered in the differential diagnosis of intraocular inflammation in areas where *F. tularensis* is endemic.

## Background

Tularemia is a zoonose caused by the bacterium *Francisella tularensis*, with most human cases being acquired after close contact with infected wild rabbits and hares, or after being bitten by an infected tick [1]. It presents as a local cutaneous eschar and lymphadenitis with some patients developing systemic clinical signs including fever, rash and pneumonia [1]. In the event of an ocular portal of entry for *F. tularensis*, tularemia may present as Parinaud’s oculoglandular syndrome consisting of conjunctivitis associated with preauricular lymphadenitis [2]. In some patients, systemic signs and symptoms correlate with the blood-borne dissemination of the organism [1] and one case of “serpiginous-like choroiditis” has been described [3], with atypical characteristics such as peripapillary sparing. However, no case of uveitis has been reported in the course of tularemia and this etiology is not routinely included in the standard examination of uveitis patients. Using an “uveitis kit” aimed at screening for all infectious causes in uveitis patients [4], we revealed epidemiological and serological evidence of tularemia as the cause of uveitis in one patient, whose case we describe here.

## Case presentation

**Fig. 1:**
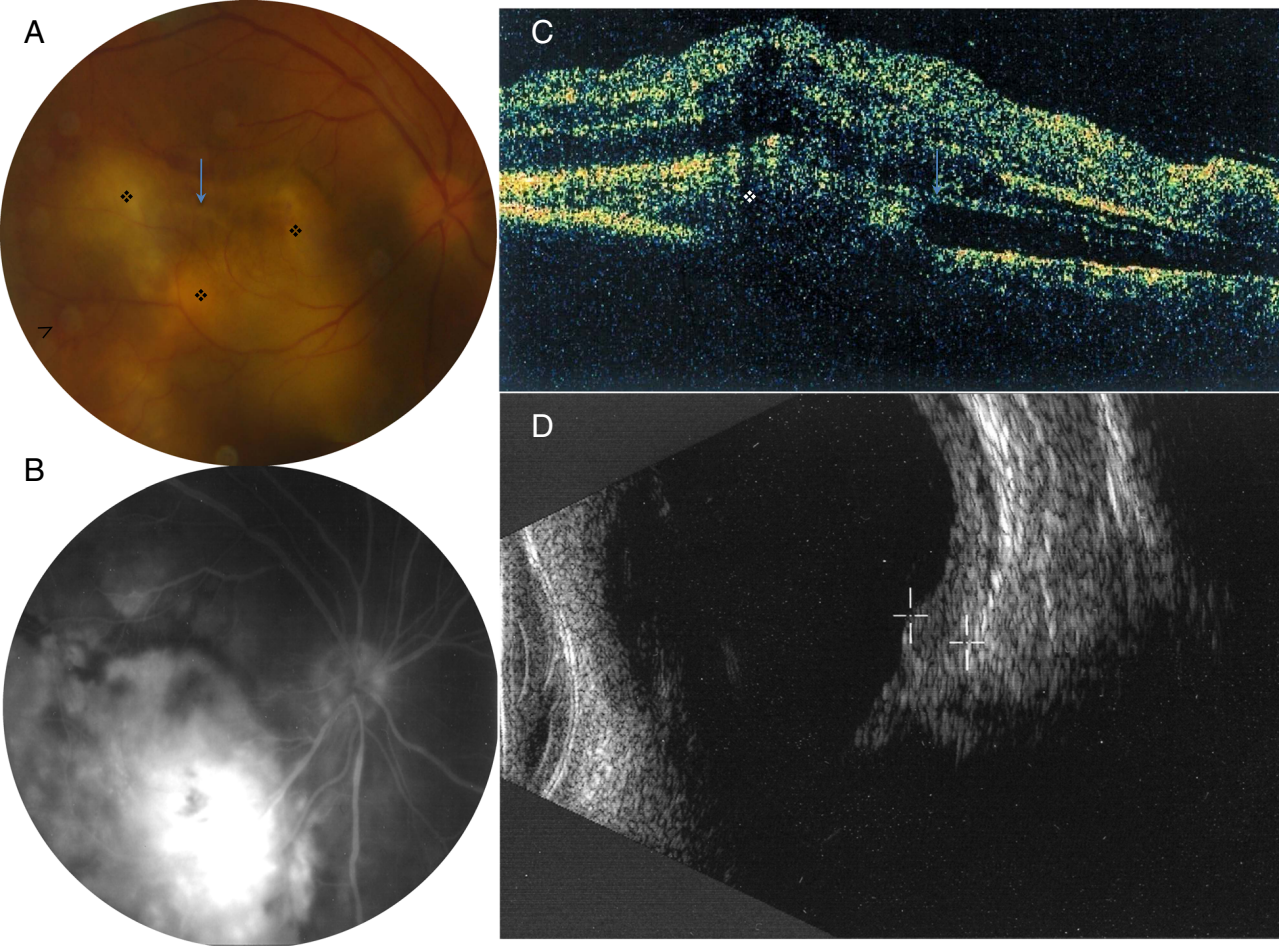
**a** Fundus examination of the right eye revealed a multifocal large yellowish choroidal infiltrate surrounding by hemorrhages (*arrow head*), associated with an overlying subretinal detachment, and subretinal fibrosis (*arrow*). **b** Fluorescein angiography showed late hyperfluorescence with focal vascular leakage. **c** OCT B scan showed choroidal infiltrates with serous retinal detachment and fibrosis (**d**) Ultrasound biomicroscopy confirmed the presence of a 3.8 mm parietal granuloma with few calcifications

In February 2008, a 52-year-old Caucasian man presented at the ophthalmology department for the diagnosis and treatment of unilateral uveitis. His past medical history was unremarkable until December 2007 when he presented with a 38 °C fever and headaches. The patient was a hunter living in northern France and he recalled having killed rabbits in the days before the symptoms appeared. Biological investigations found elevated C-reactive protein levels at 40 g/L and cerebrospinal fluid investigations eliminated meningitis. Three weeks later the patient suffered floaters and loss of vision in the eye. Visual acuity was 20/20 in the left eye and finger counting with the right eye. Biomicroscopy disclosed a mild anterior reaction with a few small keratic precipitates and a 1+ flare with a few cells but without any posterior synechaie. The uveitis was posterior with a 2+ vitritis and a multifocal large yellowish choroidal infiltrate involving the macula associated with an overlying subretinal detachment, inferior intraretinal exsudates along vessels, subretinal hemorrhages and subretinal fibrosis (Fig. 1). Fluorescein angiography revealed early hypofluorescence of the deep infiltrate and progressive late hyperfluorescence with focal vascular leakage. Deep hemorrhages masked fluorescence during all frames. OCT confirmed subretinal fluid, hyper-reflective fibrosis and heaped-up cells anterior to the pigment epithelium. Ultrasound biomicroscopy confirmed the presence of a 3.8 mm Choroidal granuloma with a few calcifications in the left eye. The initial work-up was negative and a neoplastic disease masquerading as an intraocular inflammation was excluded. After the diagnosis was reached as reported below, the patient declared that several cases of tularemia had been diagnosed among those of his relatives who were hunters. Uveitis was rapidly controlled after specific antibiotic therapy (doxycycline, 200 mg a day for three weeks) with a subretinal fibrotic scar and complete resolution of subretinal fluid. Final visual acuity remained limited to counting fingers. During telephone interview three years after starting the treatment, the patient still complained of a visual loss with central scotoma.

An anterior chamber puncture and a serum specimen were collected as part of a “uveitis kit” for standardized laboratory tests, as previously described [4]. These investigations including molecular and serological searches for intracellular bacteria comprising *Rickettsia* spp., *Coxiella burnetii, Bartonella* spp. and *Tropheryma whipplei* remained negative except for an indirect immunofluorescence assay for *F. tularensis* which yielded IgG 400 and IgM 50. In order to further confirm the specificity of this serological reaction, we performed two-dimensional electrophoresis (2-DE)-immunoblotting and mass spectrometry using a *F. tularensis* subsp. *holarctica* strain URFT1 isolated in cell-culture in our laboratory as the antigen [5]. Purified bacteria in phosphate buffered saline (PBS), supplemented with protease inhibitors, were disrupted by sonication and clarified by centrifugation (12,000 × *g*, 4 °C, 10 min). Soluble proteins were precipitated and solubilised. Iso-electro-focalization was performed according to the manufacturer’s protocol (Multiphor II, Pharmacia) using Immobiline DryStrips (13 cm, pH 3 to 10, Amersham). After two equilibrations, the strips were embedded in 0.5 % agarose and the proteins were resolved using 11.25 % sodium dodecylsulfate (SDS) polyacrylamide gel electrophoresis (SDS-PAGE). Proteins were visualized using silver staining and were transferred onto nitrocellulose membrane. Polyacrylamide gels were also stained with silver stain to check the quality of migration. Blocked membranes were incubated for two hours with the patient’ serum (dilution 1:500) or with a healthy blood donor’s serum as a negative control. Thus treated, the membranes were washed three times with PBS-Tween and incubated with peroxidase-conjugated immunoglobulin goat anti-human antibody1:1000 (Southern Biotechnology, Birmingham, Alabama, USA) for one hour. The membrane was then washed three times, as indicated above. Immunostained spots were visualized using a commercially available chemiluminescence kit (ECL Western Blotting Analysis System, GE Healthcare). The membranes were then exposed to hyperfilm ECL (GE Healthcare) and subsequently developed using an automated film processor (Hyperprocessor, GE Healthcare). For identification of proteins by matrix-assisted laser desorption-ionisation time-of-flight mass spectrometry, spots were excised from gels and stored at −80 °C.

We tested the serum of the tularemia patient on the *F. tularensis* whole cell extract (separated by 2-DE using broad range IPG strips of pH 3–10). The silver stained gel was used as a reference map and other gels were used for Western blot. The pattern of immunoreactivity of the tularemia serum revealed 81 reactive spots all identified by mass spectrometry as being specific for *F. tularensis*; Nine spots (11 %) were also detected by the negative control serum and were identified as GroEL chaperone, elongation factor TU, 30S ribosomal protein S1, phosphogluconomutase, succinate dehydrogenase, 3-oxoacyl-[acyl-carrier protein] synthase III and NADP-specific glutamate dehydrogenase.

## Conclusions

In this patient, the only evidence gathered from a standardized laboratory procedure aimed at reaching an exhaustive diagnosis of infectious uveitis was *F. tularensis* [4]. The diagnosis was based upon an indirect immunofluorescence serology and a new generation, two-dimensional Western-blot test and mass spectrometry analysis. We later confirmed the specificity of patient’s serum antibodies, as 89 % of peptidic spots were not detected by the negative control and peptidic spots specifically detected by patients’ serum had previously been found in proteomic analysis of *F. tularensis* [6]. In this paper, they have been confirmed as being *F. tularensis* proteins. Unsurprisingly, cross-reactive proteins included chaperone and ribosomal proteins which were already known to support a non-specific reaction in serology tests. Likewise, false positive quantiferon results have already been reported in the course of uveitis [7].

This report adds uveitis to the list of ophthalmologic manifestations of tularemia. To date, Parinaud’s oculoglandular syndrome has been the only ophthalmologic complication reported in the course of tularemia [2]. One case of serpiginous choroiditis (a type of posterior uveitis) has also been reported. The fact that molecular analysis did not detect the presence of *F. tularensis* in the ocular specimen may be due to a lack of sensitivity of the test in relation to the small quantity of material. Alternatively, it may indicate the absence of the pathogen into the ocular specimen. Such a situation has been well-described in Q fever uveitis, due to *C. burnetii*, an intracellular organism closely related to *F. tularensis* [8, 9].

Tularemia should be considered in the differential diagnosis of uveitis in areas where *F. tularensis* is prevalent. The serum of patients presenting with uveitis without first-line diagnosis should be tested for the presence of anti-*F. tularensis* antibodies.

## Abbreviations

2-DE: Two-dimensional electrophoresis
PAGE: Polyacrylamide gel electrophoresis
PBS: Phosphate buffered saline
SDS: Sodium dodecylsulfate
